# Innervated Pedicled Gracilis Flap for Dynamic Abdominal Wall Reconstruction

**DOI:** 10.1097/GOX.0000000000001852

**Published:** 2018-09-06

**Authors:** Tarik Mujadzic, Charles A. Gober, David B. Nahabedian, Edmond F. Ritter, Mirsad Mujadzic

**Affiliations:** * Augusta University, Augusta GA 30912; † University of South Carolina School of Medicine, Columbia, SC 29209.

## Abstract

Abdominal wall reconstruction for full-thickness defects is a challenging procedure that usually requires the use of flaps. The gracilis flap is known to be used in hernia repair in which the abdominal muscles are still intact, but there are no reports in literature describing the use of an innervated gracilis for dynamic abdominal wall reconstruction after tissue loss due to severe trauma. We present a surgical technique in which the gracilis is harvested preserving the neurovascular pedicle, then tunneled underneath the adductor longus to cover the lower abdominal defect and provide it with basal muscle tone without tension on the pedicle. This results in restored integrity of the musculofascial abdominal wall and dynamic muscle function and support. The gracilis flap has been proven to be useful and versatile in reconstructive surgery with great potential in abdominal wall reconstruction having minimal donor-site morbidity and hernia recurrence risk.

## Abstract 2

Supplemental Digital Content is available in the text.

## INTRODUCTION

Full-thickness defects of the abdominal wall present a difficult reconstructive challenge. Goals of abdominal wall defects reconstruction are primarily restoration of integrity of the musculofascial and overlying soft-tissue layers and provision of dynamic muscle support.^1^ Conventional approaches, such as a skin graft, local and regional flaps are usually sufficient to achieve first goal.^2^ Providing dynamic abdominal wall support can be achieved with pedicled innervated tensor fascia lata or rectus femoris pedicled flap in lower abdominal wall defect. For abdominal wall defects, above umbilicus, innervated free flaps are effective technique, however, requiring microsurgical skills and significantly longer operative time than conventional techniques.

Aim of our study was to present simple and technically easy idea for dynamic abdominal wall reconstruction.

## PATIENTS AND METHODS

**Fig. 1.**
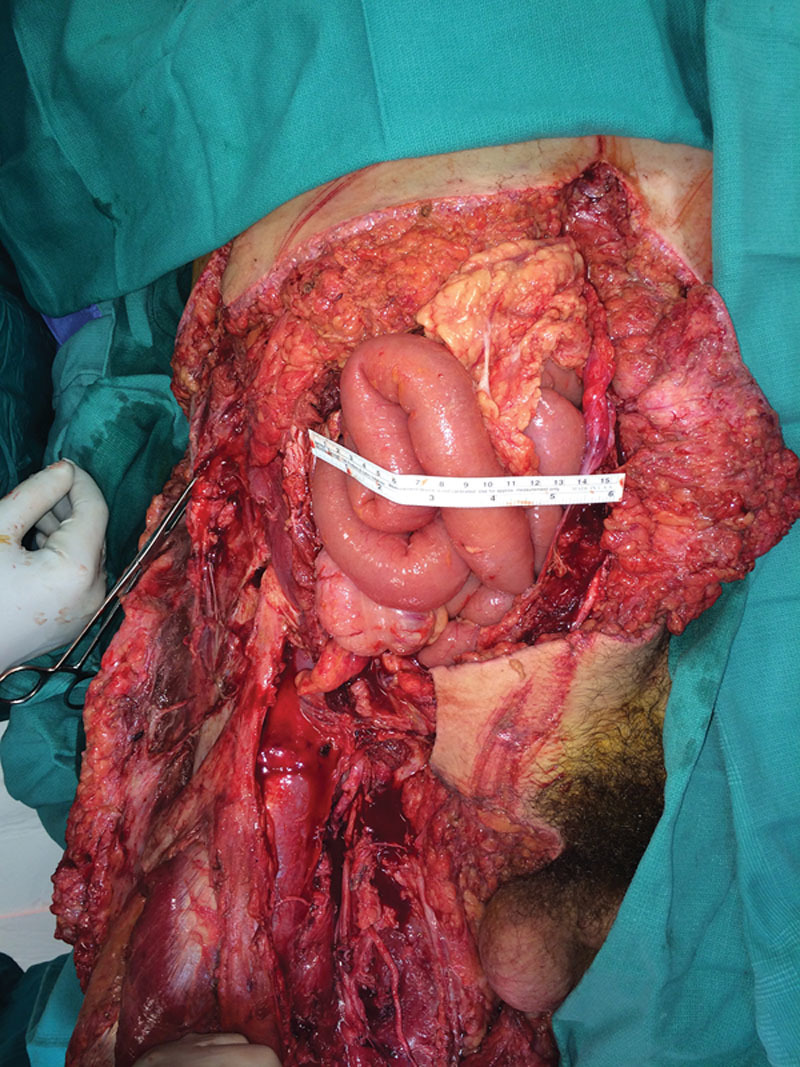
Initial presentation showing the loss of abdominal tissue and eviscerated bowels in the right lower quadrant and deep laceration of anterior right thigh exposing the femoral vessels and nerve.

A 22-year-old male presents as a Level 1 trauma following a work-related injury. He was caught under a ditch witch causing a large complex laceration spanning from the right lower quadrant of his abdomen down to just below his right knee with avulsion of skin, muscle, tendon, fascia, and evisceration of the bowel in the right lower quadrant of abdomen. The quadriceps and distal portion of the tensor fascia lata were torn and shredded. The patient suffered full-thickness loss of the musculature in the right lower abdomen, including the rectus abdominis and part of the obliques. The neurovascular bundle in the femoral triangle was exposed and the femoral nerve was stretched and transected. Branches of the femoral nerve going into the rectus femoris, vastus lateralis, and vastus medialis were avulsed (Fig. 1). However, the main trunk of the femoral nerve was intact and the femoral artery and vein. Ipsilateral leg sensation and motor function of the foot and ankle were preserved. To reconstruct and close the abdominal wall defect, the trauma team initially closed the abdomen with a bridging alloderm patch (See figure, Supplemental Digital Content 1, which displays a sketch of the patient after initial closure of abdomen with Alloderm patch by trauma service. http://links.lww.com/PRSGO/A812). The plastic surgery team then mobilized the pedicled tensor fascia lata medially to cover the exposed femoral vessels and to reconstruct the inguinal ligament and inferior portion of the abdominal wall. A wound vac was placed over the alloderm in the full abdominal wall defect. In the next procedure, we used the pedicled gracilis flap to cover the full-thickness abdominal defect.

### Surgical Technique

**Fig. 2.**
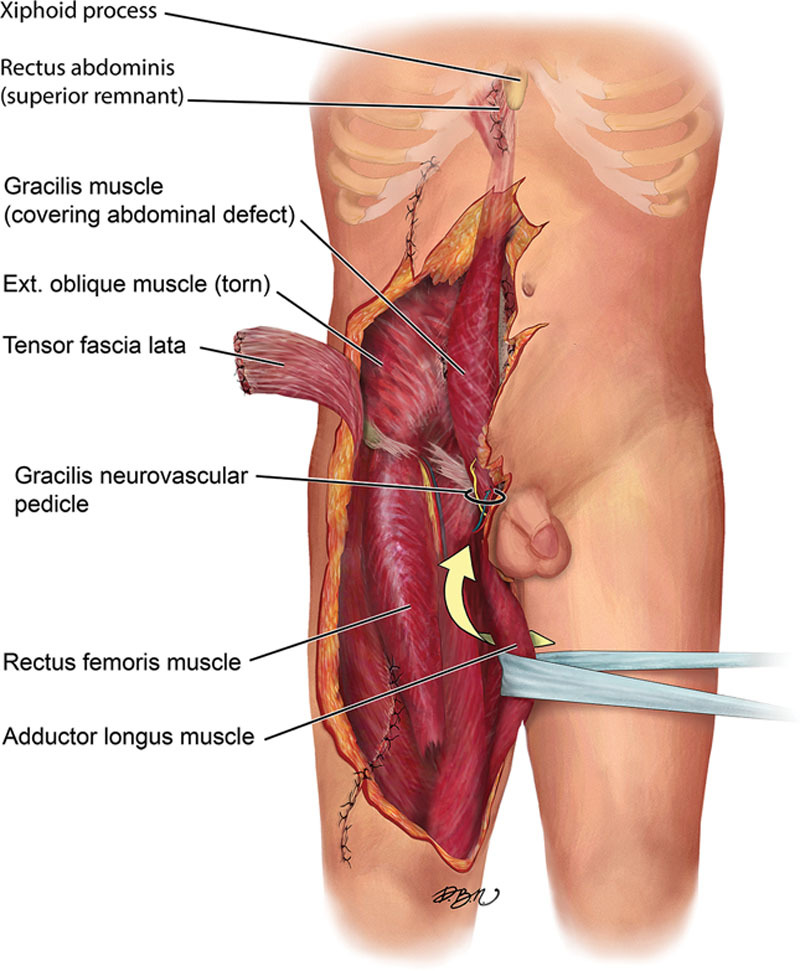
Mobilization of the gracilis underneath the adductor longus after completely detaching it from its origin and insertion.

**Fig. 3.**
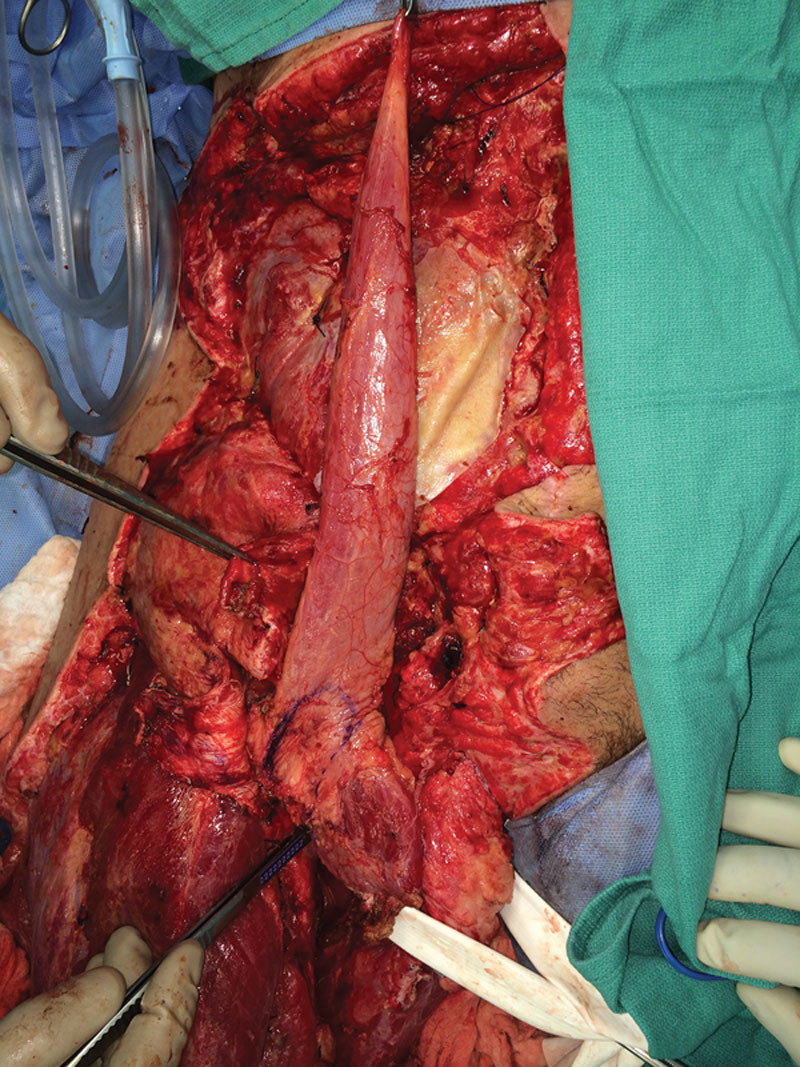
Mobilization of the gracilis underneath the adductor longus after completely detaching it from its origin and insertion.

**Fig. 4.**
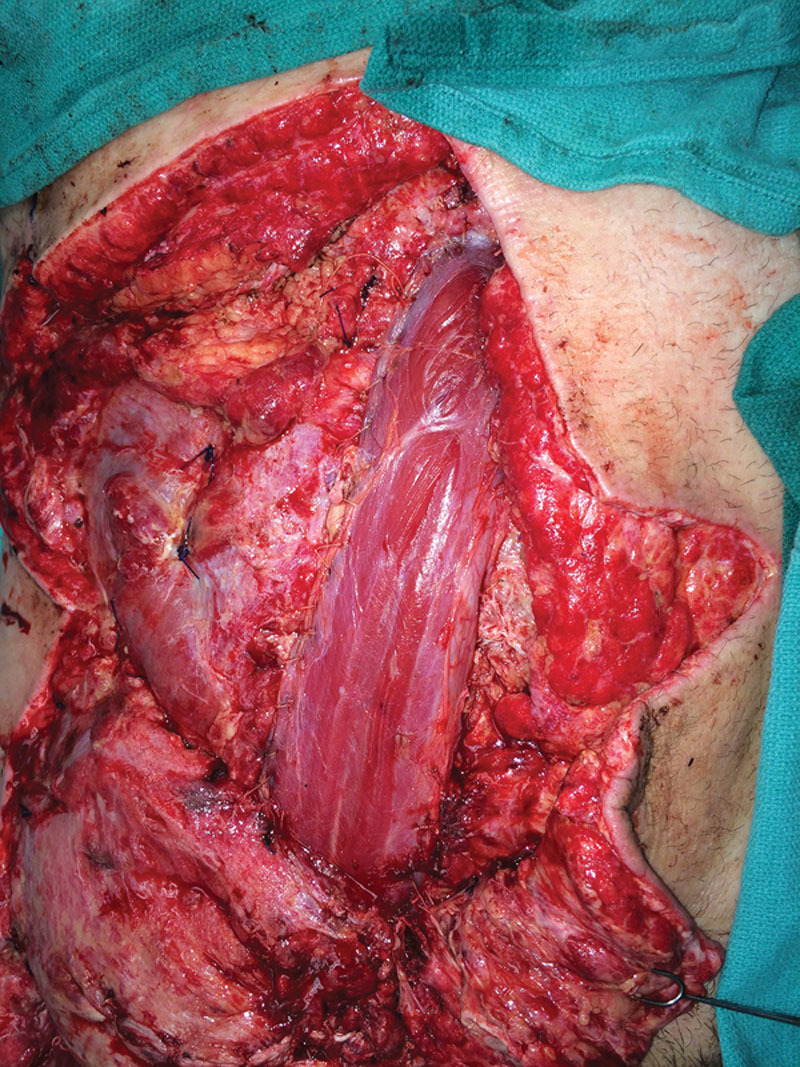
The gracilis in place tensed over the defect sutured to the remnants of the abdominal muscle fascia on the sides. Here, we also see the tensor fascia lata covering the femoral neurovascular bundle and providing support in place of the inguinal ligament.

The pubic attachment of the gracilis was completely detached subperiosteally, and the muscle insertion point just below the medial condyle of the tibia was also detached. The full length of the tendon was preserved. The vascular pedicle was fully dissected and freed to the profunda femoris, and the anterior obturator nerve was separately dissected and mobilized. The pedicled flap was then passed underneath the adductor longus and inverted so that the original distal end reached the xiphoid process (Figs. 2, 3). The pubic origin was then sutured to the pubic periosteum at the adductor longus origin. The gracilis tendon was sutured to the superior remnant of the rectus abdominis muscle. The fascia of the oblique abdominal muscles laterally and rectus abdominis medially were pulled in and stitched to the gracilis fascia on the sides. Using the innervated gracilis muscle, the torn rectus abdominis muscle and full-thickness defect in the abdominal wall musculature was reconstructed spanning the length of the right abdomen (Fig. 4; See figure, Supplemental Digital Content 2 which displays a sketch of the patient after Gracilis flap was inset in abdominal defect. http://links.lww.com/PRSGO/A813). Subsequently, the patient was skin grafted and has since healed well. On 18-month follow-up, he had no signs of herniation, and there were visible gracilis muscle contractions (See figure, Supplemental Digital Content 3 which displays a AP view of patient abdomen 18 months after original surgery. http://links.lww.com/PRSGO/A814; See figure, Supplemental Digital Content 4 which displays a profile view of patient abdomen 18 months after original surgery. http://links.lww.com/PRSGO/A815).

## DISCUSSION

There are many different techniques used in abdominal wall reconstruction. In large hernia defects where the abdominal muscles are intact, component separation technique and abdominal wall reconstruction with or without the use of biologic or synthetic mesh are commonly used and have been described.^3^ However, in cases where part of the abdominal wall is missing either secondary to tumor resection, infection, or trauma, a different approach is necessary. In these situations, local, pedicle, or free flap reconstruction typically must be used. The algorithm for abdominal wall repair provides us with several options such as using synthetic mesh, component separation technique, and flaps.^1,2^ Given the severity of the defect, flaps would be appropriate for repair. Commonly used flaps are the rectus femoris or tensor fascia lata usually used to just cover the defect without dynamic support. However, avulsion of tissue in the right lower abdomen and right anterior thigh left the gracilis as the only viable option in providing dynamic abdominal support. The gracilis is a very versatile flap that is an excellent choice for its ease of harvesting and minimal donor-site morbidity.^4,5^ Pedicled gracilis flaps have been routinely used in reconstructing perineal defects and for vaginal reconstruction.^6^ In our literature search, there was only 1 study on the use of an innervated gracilis for abdominal wall hernia repair described by Venugopalan^7^ in 1980. The flap was used to repair a midline abdominal incisional hernia in 20 patients with 0% recurrence rate. However, we were unable to find cases where the innervated gracilis was used for abdominal repair in a posttraumatic full-thickness wall defect with tissue loss, such as the 1 being presented.

The technique by Venugopalan^7^ describes the gracilis only being able to reach the level of the umbilicus when stretched proximally to cover the lower abdominal defect. Our technique differs in that we detached the gracilis at both the origin and insertion site, allowing us to mobilize the muscle underneath the adductor longus and reaching as high up as the xiphoid process, thus achieving considerably more length. Unlike in Venugopalan’s technique where no tension was allowed due to potential kinking and compromise of vascular supply, we fully mobilized the pedicle allowing reattachment of the gracilis origin and placing the muscle into basal tension by attaching it between the 2 aforementioned insertion and origin points. This provides restitution of dynamic abdominal wall reconstruction. Although in this case we didn’t harvest the full extent of the gracilis fascia, it’s possible to harvest fascia of the adjacent adductor longus and adductor magnus to strengthen the repair or if a fascial defect is present.

## CONCLUSIONS

We believe that pedicled gracilis flap in selective cases may be good alternative for free neurotized flap for abdominal wall reconstruction. Surgery is technically easier and operative time is much shorter than free flap. Our experience with this technique suggests that it might be a worthwhile modality for dynamic reconstruction for moderate size of abdominal defects.

## Supplementary Material
