# Supplementary material for: Innervated Pedicled Gracilis Flap for Dynamic Abdominal Wall Reconstruction
Source: Plast Reconstr Surg Glob Open. 2018 Sep 6;6(9):e1852. doi: 10.1097/GOX.0000000000001852 (PMC6191209; doi:10.1097/GOX.0000000000001852)
Supplement: Supplementary file 1 [file gox-6-e1852-s001.pdf]

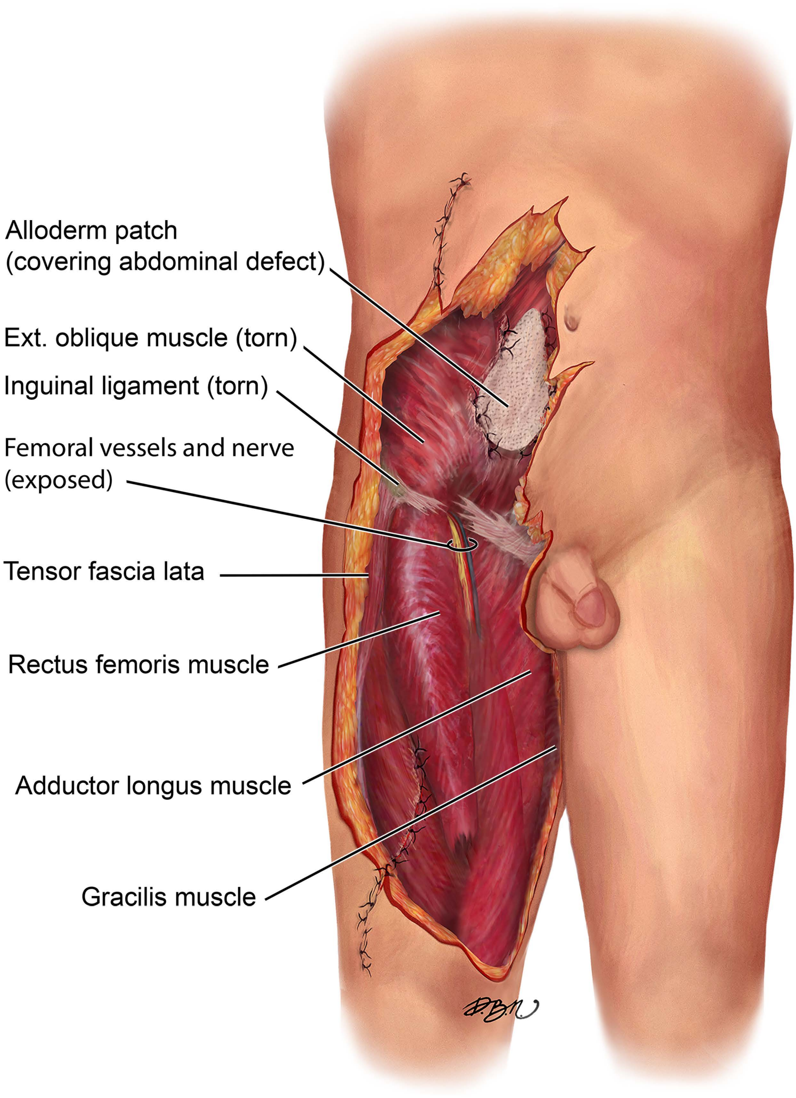
Alloderm patch
(covering abdominal defect)
Ext. oblique muscle (torn)
Inguinal ligament (torn)
Femoral vessels and nerve
(exposed)
Tensor fascia lata
Rectus femoris muscle
Adductor longus muscle
Gracilis muscle
