# Supplementary material for: Innervated Pedicled Gracilis Flap for Dynamic Abdominal Wall Reconstruction
Source: Plast Reconstr Surg Glob Open. 2018 Sep 6;6(9):e1852. doi: 10.1097/GOX.0000000000001852 (PMC6191209; doi:10.1097/GOX.0000000000001852)
Supplement: Supplementary file 2 [file gox-6-e1852-s002.pdf]

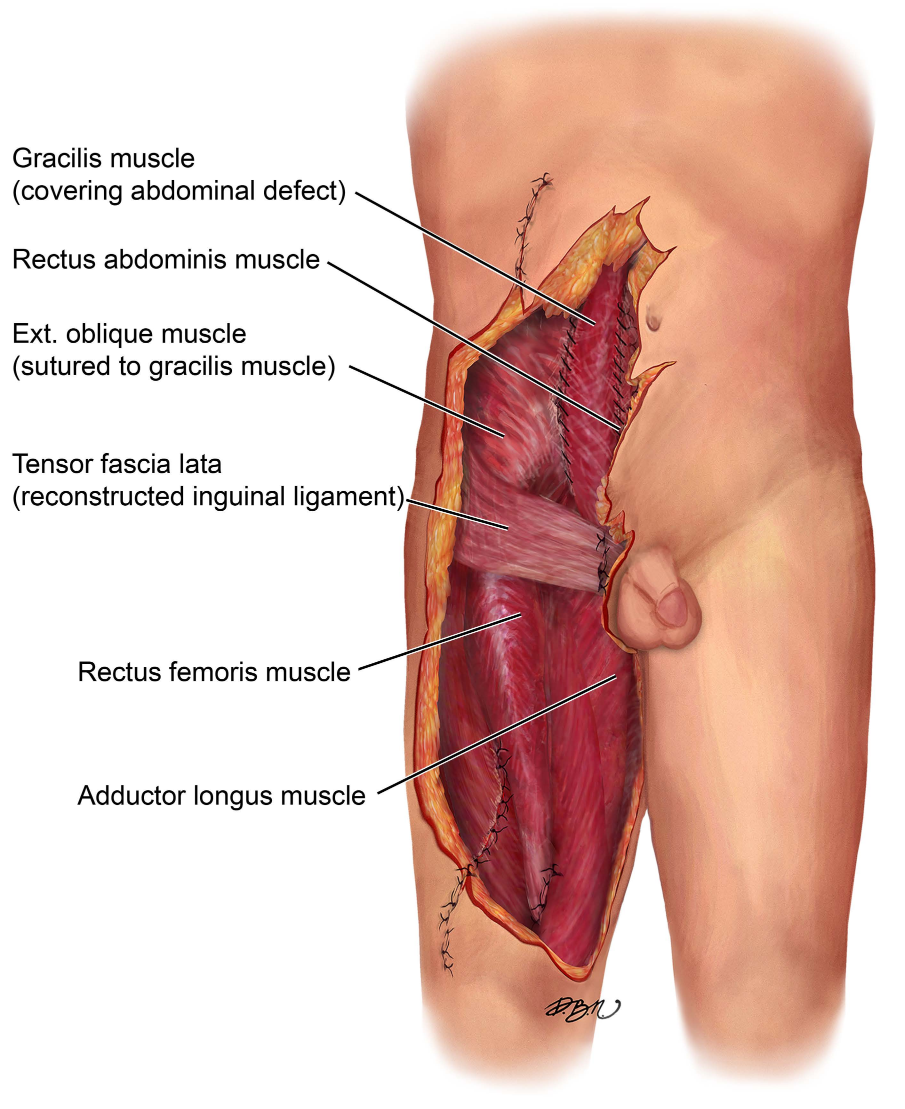

Gracilis muscle
(covering abdominal defect)
Rectus abdominis muscle
Ext. oblique muscle
(sutured to gracilis muscle)
Tensor fascia lata
(reconstructed inguinal ligament)
Rectus femoris muscle
Adductor longus muscle
