# Supplementary figures and images for: Innervated Pedicled Gracilis Flap for Dynamic Abdominal Wall Reconstruction
Source: Plast Reconstr Surg Glob Open. 2018 Sep 6;6(9):e1852. doi: 10.1097/GOX.0000000000001852 (PMC6191209; doi:10.1097/GOX.0000000000001852)

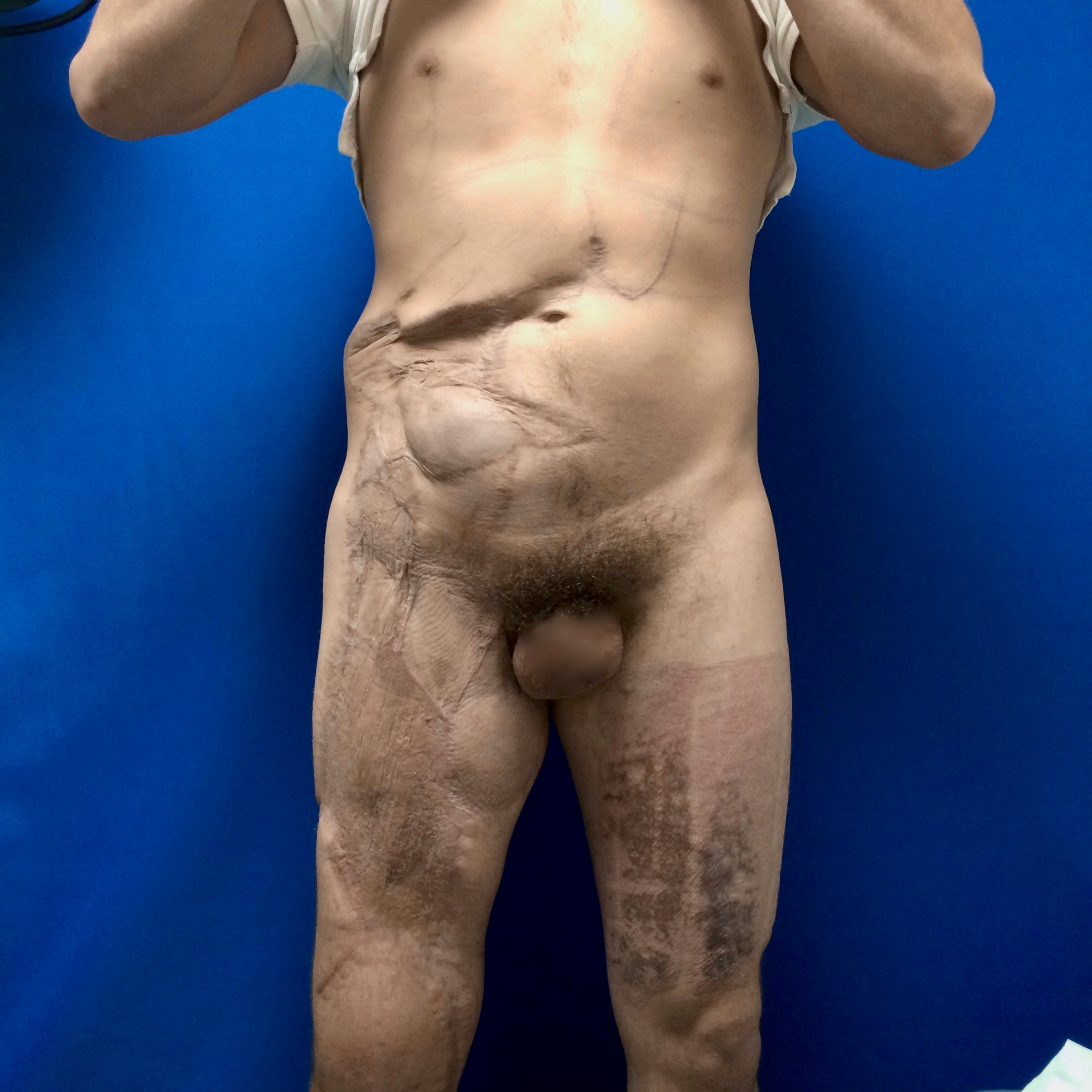

Supplement: Supplementary file 3 [file gox-6-e1852-s003.pdf]

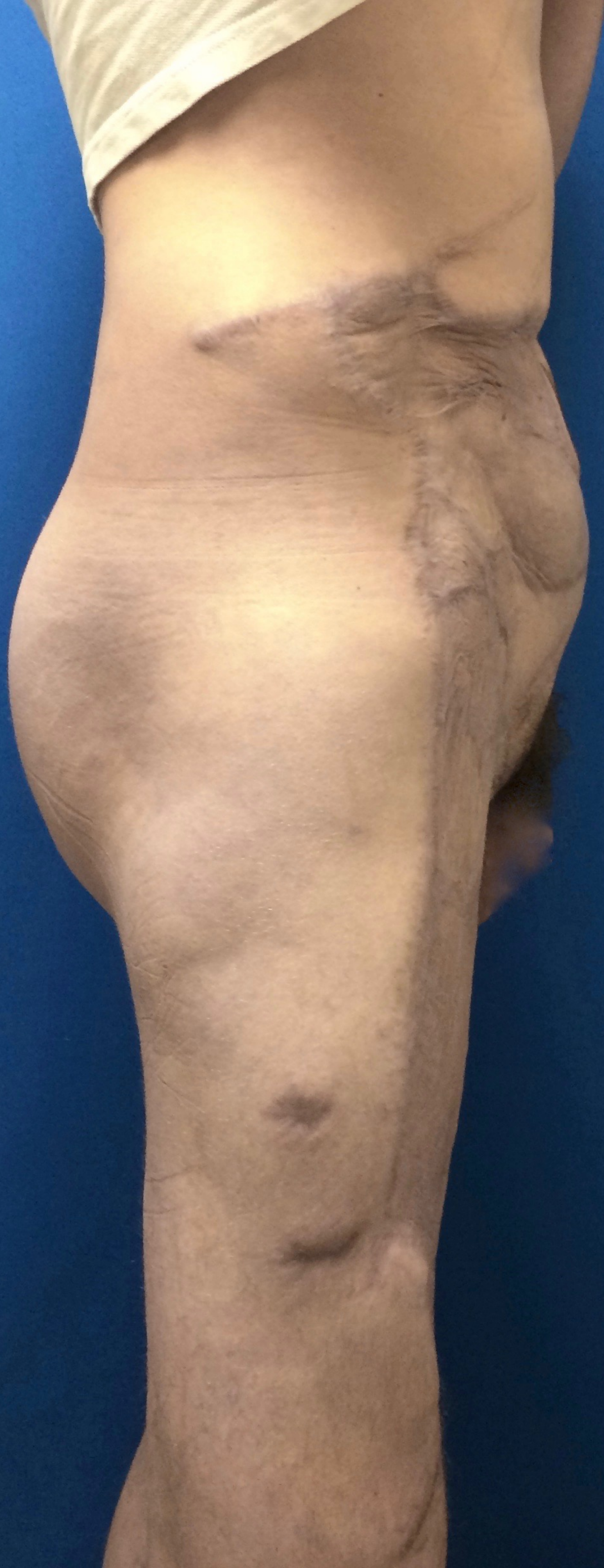

Supplement: Supplementary file 4 [file gox-6-e1852-s004.pdf]
